# Estimating stomatal conductance of citrus under water stress based on multispectral imagery and machine learning methods

**DOI:** 10.3389/fpls.2023.1054587

**Published:** 2023-02-10

**Authors:** Jiaxing Xie, Yufeng Chen, Zhenbang Yu, Jiaxin Wang, Gaotian Liang, Peng Gao, Daozong Sun, Weixing Wang, Zuna Shu, Dongxiao Yin, Jun Li

**Affiliations:** ^1^ College of Electronic Engineering (College of Artificial Intelligence), South China Agricultural University, Guangzhou, China; ^2^ Guangdong Laboratory for Lingnan Modern Agriculture, South China Agricultural University, Guangzhou, China; ^3^ Department of Mechanical and Electrical Engineering, Luoding Polytechnic, Yunfu, China; ^4^ College of Engineering, South China Agricultural University, Guangzhou, China

**Keywords:** stomatal conductance, threshold segmentation, VI, GLCM, machine learning

## Abstract

**Introduction:**

Canopy stomatal conductance (Sc) indicates the strength of photosynthesis and transpiration of plants. In addition, Sc is a physiological indicator that is widely employed to detect crop water stress. Unfortunately, existing methods for measuring canopy Sc are time-consuming, laborious, and poorly representative.

**Methods:**

To solve these problems, in this study, we combined multispectral vegetation index (VI) and texture features to predict the Sc values and used citrus trees in the fruit growth period as the research object. To achieve this, VI and texture feature data of the experimental area were obtained using a multispectral camera. The H (Hue), S (Saturation) and V (Value) segmentation algorithm and the determined threshold of VI were used to obtain the canopy area images, and the accuracy of the extraction results was evaluated. Subsequently, the gray level co-occurrence matrix (GLCM) was used to calculate the eight texture features of the image, and then the full subset filter was used to obtain the sensitive image texture features and VI. Support vector regression, random forest regression, and k-nearest neighbor regression (KNR) Sc prediction models were constructed, which were based on single and combined variables.

**Results:**

The analysis revealed the following: 1) the accuracy of the HSV segmentation algorithm was the highest, achieving more than 80%. The accuracy of the VI threshold algorithm using excess green was approximately 80%, which achieved accurate segmentation. 2) The citrus tree photosynthetic parameters were all affected by different water supply treatments. The greater the degree of water stress, the lower the net photosynthetic rate (Pn), transpiration rate (Tr), and Sc of the leaves. 3) In the three Sc prediction models, The KNR model, which was constructed by combining image texture features and VI had the optimum prediction effect (training set: R^2^ = 0.91076, RMSE = 0.00070; validation set; R^2^ = 0.77937, RMSE = 0.00165). Compared with the KNR model, which was only based on VI or image texture features, the R^2^ of the validation set of the KNR model based on combined variables was improved respectively by 6.97% and 28.42%.

**Discussion:**

This study provides a reference for large-scale remote sensing monitoring of citrus Sc by multispectral technology. Moreover, it can be used to monitor the dynamic changes of Sc and provide a new technique for gaining a better understanding of the growth status and water stress of citrus crops.

## 1 Introduction

Citrus fruits are some of the most important fruits in the world and are mainly distributed between the latitudes of approximately 30° N and 30° S (Liu et al., 2022). Citrus fruits are mainly grown in China, the United States, and India (Zhang et al., 2021b). Since 2007, China’s citrus planting area and output have been ranked first in the world. According to the National Bureau of Statistics of China, the area of citrus cultivation in China increased from 1,061,200 hm^2^ in 1990 to 2,617,300 hm^2^ in 2019. Moreover, the total output has increased almost tenfold, from 4,854,900 T in 1990 to 45,845,400 T in 2019 (Wan et al., 2021). However, problems such as uneven quality and the unstable yield of citrus fruits are becoming increasingly serious, which affects the sustainable development and export levels of the Chinese citrus industry (Tu et al., 2021). Therefore, how to judge and improve the quality of citrus fruits is the key to industrial development. Climate change, desertification, and the overexploitation of water resources due to overpopulation and agricultural intensification will challenge the survival, growth, and yield of agricultural commodities. Especially in citrus crops, water scarcity can negatively affect cell metabolism, overall tree growth, and fruit quality. Moreover, drought stress can affect the postharvest treatment of citrus fruits, rendering them more vulnerable to damage during handling and transportation (Carr, 2012). The leaves of citrus fruits are the most sensitive organ to water stress. After more than 4 hours of water stress, leaf rolling is evident. In addition, when the root is short of water, the outer epidermis is damaged and the cell shape changes. Other effects of a lack of water are a cessation of shoot growth and early maturity. Severe water shortages can produce irreversible damage to growth and development, even death (Miranda et al., 2022; Xie et al., 2022). Traditionally, crop water stress has been determined by *in situ* plant measurements, soil water content, and meteorological variables (Brewer et al., 2022).

Ahumada-Orellana et al. (Ahumada-Orellana et al., 2019) revealed that when the water supply to the soil of olive trees decreased, the leaves would gradually close their stomata to reduce water loss caused by transpiration. Simultaneously, this stomatal closure would also reduce carbon dioxide diffusion into the leaves, affecting the photosynthesis of crops. Zhou et al. (Zhou et al., 2021a) revealed that the stomatal conductance (Sc) of winter wheat changed under different water treatments and the magnitude of stomatal conductance was negatively correlated with the degree of water stress. However, traditional methods for measuring the Sc of citrus are time-consuming, laborious, and poorly representative. Moreover, the results of fixed-point determination do not always reflect the actual situation of citrus crops in the whole region (Huang et al., 2021; Weng et al., 2021). The use of unmanned aerial vehicle (UAV) has proved to be an effective method for detecting crop water stress and remote sensing to retrieve crop Sc measurements, which can avoid the problems of low efficiency, high costs, and difficult field operation (Ezenne et al., 2019).

Currently, the research and application of multi-spectral imaging for monitoring crop growth information are gradually increasing (Gallardo-Salazar and Pompa-García, 2020; Landi et al., 2020; Rossiter et al., 2020). Zhou et al. (Zhou et al., 2021a) used the vegetation index(VI) and image texture features to analyze the water stress of winter wheat and constructed an Sc prediction model of winter wheat under such stress. Singhal et al. (Singhal et al., 2019) combined spectral data and true surface chlorophyll to simulate chlorophyll estimation and used different machine learning algorithms for comparative analyses. Chea et al. (Chea et al., 2020) used multispectral images to calculate sugarcane canopy reflectance and constructed a sugarcane growth prediction model. Reisi Gahrouei et al. (Reisi Gahrouei et al., 2020) used multispectral data and machine learning methods to estimate the biomass and leaf area index (LAI) of cash crops. Hussain et al. (Hussain et al., 2020) used spectral VI at different resolutions to conduct quantitative analyses of rapeseed in different periods and constructed a non-destructive testing model for rapeseed canopy variables. Qi et al. (Qi et al., 2020; Qi et al., 2021) retrieved the LAI and chlorophyll content of peanuts by using the multispectral VI of peanuts with different planting densities. The results indicated that UAV multispectral VI could quickly obtain field information by relying on high-precision prediction models.

Although the accuracy of the results from these studies was acceptable, an increase in variety of input of variables could improve the accuracy of the models further (Khaliq et al., 2018; Fu et al., 2020; Zhou et al., 2020). Cheng et al. (Cheng et al., 2020) determined the LAI of maize based on VI, crop models, and data assimilation methods, achieving improved accuracy compared to any single method. Shu et al. (Meiyan et al., 2022) constructed a prediction model for the aboveground biomass of maize in multiple growth periods by combining multispectral and UAV digital images with maize LAI and plant height. To realize the prediction of soil water content, Gu et al. (Gu et al., 2021) used multispectral and thermal infrared images to determine soil water content and then constructed a temperature vegetation dryness index by combining the obtained canopy temperature and VI. Liu et al. (Liu et al., 2019) combined VI and texture features to determine the aboveground biomass of winter rape, allowing the analysis of which input features were the most important and successful predictions of the aboveground biomass for the next year.

The reliability and popularity of using spectral imaging technology for crop nutrition diagnosis are limited because the spectral reflection features of the plant canopy are affected by many factors, such as canopy geometry, soil cover, and weather conditions. Therefore, eliminating such disturbances as soil and weeds is key to establishing a universal and high-precision plant nutrition diagnosis method (Mo et al., 2021; Zhou et al., 2021b). Tang et al. (Tang et al., 2022) proposed a dark channel filtering method for 25 channels of the developed equipment to effectively eliminate the scattered light interference of the wheat canopy in spectral images. The results indicated that background segmentation could effectively eliminate background interference. Cao et al. (Cao et al., 2021) proposed a wheat lodging extraction method based on the watershed algorithm combined with the adaptive threshold segmentation hybrid algorithm. This method reduced the influence of noise and achieved an extraction accuracy of 93.58%, providing a new concept for the efficient acquisition of crop lodging resistance data. The previously mentioned methods were mainly focused on field crops (such as wheat and corn), and there are few studies on mountain orchard crops (such as citrus fruits) in southern China. Therefore, based on previous studies, we further explore the relationship between citrus fruit leaf growth information and spectral features and establish an prediction model in this paper.

In this study, citrus crops (during the rapid fruit expansion period and fructescence) under different water treatments in South China were selected as the research objects. The main research content was as follows: 1. use UAV multispectral images to judge the water stress of citrus crops; 2. determine citrus leaf image segmentation and conduct comparisons; 3. evaluate the role of VI and texture features for monitoring the Sc of citrus crops; 4. use machine learning methods, including support vector regression (SVR), random forest (RF), and k-nearest neighbor regression (KNR), to establish the citrus Sc prediction model.

The remainder of the paper comprises four sections. In Section 2, we describe the materials and methods, followed by a presentation of the results of each experiment in Section 3. In Section 4, we discuss the advantages and shortcomings of the study, followed by the conclusions and prospects in Section 5.

## 2 Materials and methods

### 2.1 Experimental location and conditions

**Figure 1:**
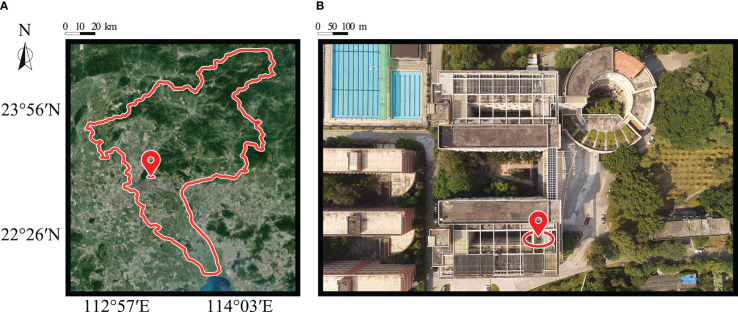
Study area: **(A)** location of South China Agricultural University in Guangzhou; **(B)** top view of the study area taken from DJI Air 2S.

The experiment was carried out in the Citrus Experimental Garden (113° 35’ E, 23° 16’ N) of the College of Engineering, South China Agricultural University, Guangzhou, Guangdong Province from October to December 2021. As shown in **Figure 1**, this region is located in the south of China with a subtropical monsoon climate, adequate solar conditions, and hot resources. The average annual sunshine is 1668.4 h and the average annual precipitation is 1793.8 mm. The experimental citrus variety was eight-year-old Citrus oleocarpa Tanaka, which was planted in pots with good growing conditions. Specimens with a similar growth status were selected as the experimental objects, and their average height was 1.73 m. The soil in the basin is sandy loam, which contains 74% sand, 19% silt, and 7% clay. The upper diameter of the basin is 0.66 m and its height is 0.36 m.

The experimental samples were divided into 4 groups labeled T1, T2, T3 and T4, with each group representing a specific water treatment. The citrus tree in T1 was supplied with sufficient water to maintain a moisture content close to field capacity (FC). While Groups T2, T3, and T4 accounted for 80%, 65%, and 50% of FC, respectively. Irrigation was performed every 2–3 days, and the amount of irrigation was 3 l, 2 l, 1 l, and 0.5 l for Groups T1–T4, respectively. The pH value of the soil layer was 6.0. To ensure uniformity of the irrigation water, regular quantitative sprinkler irrigation was used. In addition, each pot was fitted with a soil moisture sensor (JXBS-J001-EC-RS, JINGXUN, China) for real-time measurements.

### 2.2 Data collection and processing

#### 2.2.1 Multispectral camera

The multispectral camera used in the experiment was a K4 Airborne multispectral imager (hereafter, K4), which could be combined freely and was triggered remotely. This camera can be used by various small- and medium-sized multi-rotor or fixed-wing UAVs to achieve the purpose of photographing target objects on the ground. Moreover, K4 has 26 kinds of spectral filters (including 450, 560, 650, 725, 808, and 940 nm), although only 560, 650, 725, and 808 nm were used in this test. A grayscale plate was used for radiometric calibration prior to data collection.

#### 2.2.2 Multispectral data acquisition and processing

The acquisition period for the multispectral image data was from October to December 2021, which included two periods of rapid fruit expansion and fructescence. The images were collected between 11:00 and 13:00 on windless clear days when the solar irradiance was strong. The K4 camera was placed 4–5 m above the crop canopy using a support, and images were taken perpendicular to the ground to obtain an orthophoto map.

The reference plates of the experimental crops were arranged to calibrate the obtained multispectral images. The multispectral remote sensing images were then extracted, registered, and synthesized using MAPIR software, which is compatible with the K4 camera. Finally, TIFF format 4-band remote sensing images were derived, which were then imported into ENVI5.3 software for interpretation. To eliminate soil interference and shadow background values, the gray values of the citrus canopy and reference plate were extracted to calculate the 4-band reflectance of the citrus canopy. The digital quantization value (DN) of the image part 1/3 decimeter away from the outer edge of the gray plate was taken, and the formula for converting the multispectral DN value of the citrus canopy into reflectivity was as follows:


(1) $$R_{1}=\frac{D_{N1}}{D_{N2}}R_{2}$$

where 6 *R*_1_ is the reflectance of the target ground object, *D*_*N*1_ is the digital quantization value of the target ground object, *D*_*N*2_ is the digital quantization value of the grayscale plate, and *R*_2_ is the reflectance of the grayscale plate.

#### 2.2.3 Determination of photosynthetic parameters of the citrus canopy

**Figure 2:**
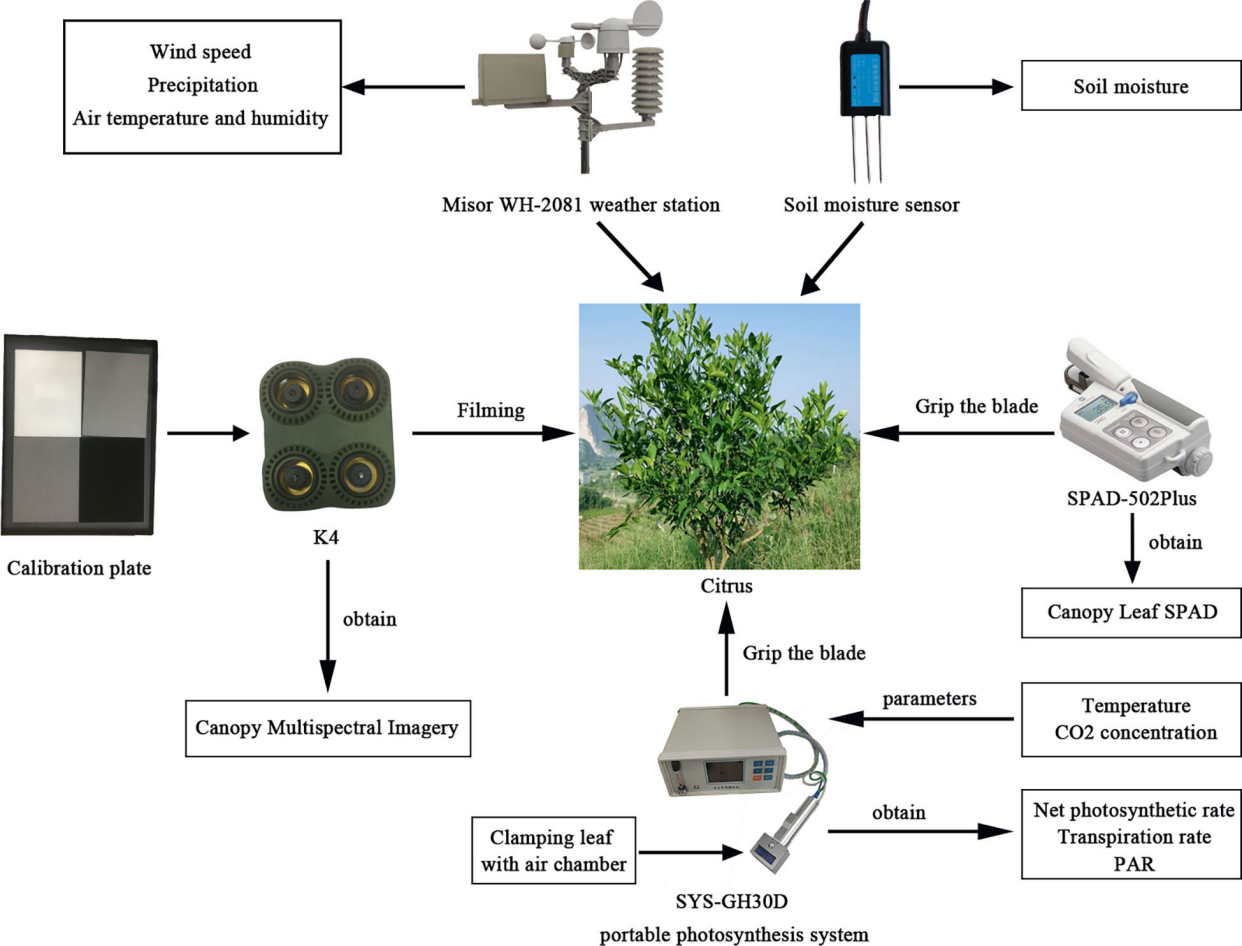
Data acquisition system.

The photosynthetic parameters of the citrus canopy were measured immediately after collection of the multispectral images. Three areas were selected for each citrus tree, and five healthy canopy leaves with good growth and no insect pests were selected for each area. The photosynthetic parameters of the citrus canopy leaves (including net photosynthetic rate (Pn), transpiration rate (Tr), Sc, and so on) were measured using an SYS-GH30D photosynthesis analyzer. The measurement range was 0–3000 µmol·m^−2^·s^−1^ with an accuracy level of 3 µmol·m^−2^·s^−1^. The average value of five leaves was used as the final determination result. **Figure 2** displays the data acquisition system. The meteorological station collected meteorological data every 30 min, 48 times a day, and the average was the average of the meteorological data during that day. The meteorological conditions during the test are displayed in **Table 1**.

**Table 1:** Meteorological data of the test area during the test.

Meteorological data	Max	Min	Average	Standard deviation
air temperature (°C)	34.60	14.00	24.12	3.45
air humidity (%)	70.00	11.00	42.56	8.56
wind speed (km/h)	15.80	0.00	2.50	3.81
precipitation (mm)	19.85	0.00	220.5(total)	/

**Table 2** presents the sample sizes of the modeling and validation sets used to construct the machine learning model and lists the statistical characteristics of the sample Sc.

**Table 2:** Stomatal conductance Sc sample data.

Dataset	Sample size	Min	Max	Average	Standard deviation
Modeling set	84	0.0080	0.0240	0.0151	0.0036
Validation set	36	0.0089	0.0245	0.0160	0.0040
Total	120	0.0080	0.0245	0.0154	0.0037

#### 2.2.4 Image segmentation algorithm of citrus canopy leaves

To realize the segmentation of citrus canopy leaves with a background of soil and pots, the effects of a global threshold segmentation algorithm and a HSV variable threshold segmentation algorithm on citrus canopy leaf image segmentation were compared. The global threshold adaptive segmentation algorithm based on maximum between-class variance (Otsu) (Xu et al., 2011) was applied to determine the gray level of the segmentation threshold by calculating the between-class variance of the background and crop objects. Subsequently, corresponding binary images were obtained. The original images were processed by different vegetation indices and compared the effect of Otsu’s algorithm on visible light VI images processed by different vegetation indices. The HSV variable threshold segmentation method was then used to segment the whole target citrus group. The values of H (Hue), S (Saturation) and V (Value) were adjusted respectively and the segmentation effect changed accordingly.

To verify the segmentation effectiveness of the algorithm, the evaluation method was based on pixels. By calculating the number of pixels of the leaf region in the manually segmented images and the number of pixels of the leaf region of the image obtained by the two segmentation algorithms, the accuracy (ACC) and Matthews’s correlation coefficient (MCC) were used to verify the segmentation accuracy of the segmentation algorithm (Al-antari et al., 2018). Here, ACC is defined as


(2) $$ACC=\frac{f_{0}}{f}\times 100\%,$$

where *f*_0_ represents the intersection between the leaf pixel region obtained by the segmentation algorithm and the leaf pixel reference region obtained by manual segmentation, and *f* represents the leaf pixel reference region obtained by manual segmentation. In addition, MCC is defined as


(3) $$MCC=\frac{T_{P}\cdot T_{N}-F_{P}\cdot F_{N}}{\sqrt{\left(T_{P}+F_{P}\right)\left(T_{P}+F_{N}\right)\left(T_{N}+F_{P}\right)\left(T_{N}+F_{N}\right)}},$$

where *T*_*P*_ represents the true positive, which is the number of overlapping pixels in the reference area of the blade obtained by manual segmentation and the foreground area of the blade obtained by the algorithm. Term *T*_*N*_ represents the true negative, which is the number of overlapping pixels in the background reference area of the image obtained by manual segmentation and the background area of the image obtained by the proposed algorithm. Term *F*_*N*_ represents the false negative, which is the reference area of the leaf foreground marked by manual segmentation. However, the number of pixels in the background area of the image is marked by the algorithm in this paper. Term *F*_*P*_ represents the false positive, which is the reference area of the image background marked by manual segmentation. However, the number of pixels in the foreground area of the leaf is marked by the algorithm in this paper. The value of MCC was in the range {−1,1}, and the larger the value, the more accurate the segmentation.

#### 2.2.5 Vegetation index selection

The VI is a dimensionless index parameter formed by a linear or nonlinear combination of the reflectance of different bands of spectral images according to the spectral absorption characteristics of vegetation (Pei et al., 2021). The VI can reflect the difference between the reflection of vegetation in visible and near-infrared bands and the soil background, which is a relatively simple and effective empirical measure of the surface vegetation condition. Seven vegetation indices were used in this study, and different spectral indices were selected to evaluate the effect of bands on the retrieval of photosynthetic parameters. Combined with previous research results, the VI selected in this study is shown in **Table 3**.

**Table 3:** Calculation formula for the vegetation index.

Vegetation index	Formula	Reference
Normalized difference VI (NDVI)	(*B*_808_-*B*_650_)/(*B*_808_+*B*_650_)	(Pei et al., 2021)
Chlorophyll VI (CVI)	*B*_808/_ *B*_560_/*B*_650_/*B*_560_	(Vincini et al., 2008)
Red-edge chlorophyll index (*CI_RE_*)	*B*_808_/*B*_725_ - 1	(Ye et al., 2021)
Green normalized difference VI (GNDVI)	(*B*_808_-*B*_560_)/(*B*_808_+*B*_560_)	(Lee et al., 2021)
Normalized Difference Greenness VI (NDGI)	(*B*_560_-*B*_650_)/(*B*_560_+*B*_650_)	(Cogato et al., 2021)
Normalized difference red edge (NDRE)	(*B*_808_-*B*_725_)/(*B*_808_+*B*_725_)	(Tavares et al., 2022)
Ratio VI (RVI)	*B*_808_/*B*_650_	(Wang et al., 2021)

#### 2.2.6 Texture feature extraction

According to the gray level co-occurrence matrix (GLCM) (Gadelmawla, 2004), eight image texture features were selected to evaluate the correlation between texture features and the photosynthetic parameters of citrus canopy leaves: mean (MEA), variance (VAR), homogeneity (HOM), contrast (CON), dissimilarity (DIS), entropy (ENT), second moment (SEC), and correlation (COR). The calculation formulae are as follows:


(4) $$MEA=\sum_{i}\sum_{j}i\times p(i,j),$$

(5) $$VAR=\sum_{i}\sum_{j}{\left(i-u\right)}^{2}p(i,j),$$

(6) $$HOM=\sum_{i}\sum_{j}\frac{1}{1+{\left(i,j\right)}^{2}}p(i,j),$$

(7) $$CON=\sum_{n=0}^{N_{g}}n^{2}\left\{\begin{array}{c} \sum_{i=1}^{N_{g}}\sum_{j=1}^{N_{g}}p(i,j)\\ |i-j|=n \end{array}\right\},$$

(8) $$DIS=\sum_{n=1}^{N_{g}-1}n\left\{\begin{array}{c} \sum_{i=1}^{N_{g}}\sum_{j=1}^{N_{g}}p(i,j)\\ |i-j|=n \end{array}\right\},$$

(9) $$ENT=-\sum_{i}\sum_{j}p(i,j)\log(p(i,j)),$$

(10) $$SEC=\sum_{i}\sum_{j}{\left\{p(i,j)\right\}}^{2},$$

(11) $$COR=\frac{\sum_{i}\sum_{j}(i,j)p(i,j)-\mu_{x}\mu_{y}}{\sigma_{x}\sigma_{y}}.$$

Here, p (i, j) is the value of the (i, j)^th^ entry in the gray level cooccurrence matrix; *N*_*g*_ is the number of distinct gray levels in the quantized image; *μ*_*x*_ and *σ*_*x*_ are the mean and standard deviation of x rows in the matrix calculation, respectively; and *μ*_*y*_ and *σ*_*y*_ are the mean and standard deviation of y rows in the matrix calculation, respectively.

#### 2.2.7 Selection of image texture features and vegetation index

The subset screening method used the whole subset regression analysis to fit and model all possible combinations of different independent variables with the least square method, and ultimately, to select an optimal monitoring model (Love, 2005). Specifically, m is the number of independent variables, and least squares fitting was performed on the x variables (x = 1, 2,…, m) in m and the dependent variables. Among all the fitting models, the best model was selected based on the full subset screening results. The evaluation criteria for selecting the optimal model were as follows: ① maximize the likelihood function; and ② minimize the unknown parameters of the model, meaning search for the best balance between model fit accuracy and model complexity. Similar to the Akaike information criterion (AIC), the Bayesian information criterion (BIC) is often used as an evaluation criterion in model selection. However, the BIC is more effective at avoiding the problem of over-complex models caused by high model accuracy. When the dimensionality is too large and the number of samples is relatively small, the BIC can also effectively avoid too many selected variables. Therefore, the principle of the smallest BIC value was observed in the selection of variables, and the leaps package of R3.5.1 software was used to conduct the full subset selection analysis. The calculation formula of BIC is as follows:


(12) BIC=kln(n)−2ln(L),

where k is the number of model parameters, n is the number of samples, and L is the likelihood function. The results of the variable screening are displayed in **Table 4**.

**Table 4:** Variable screening statistics based on full subset selection.

Type	Parameter	Number	Screening result	BIC
VIs	B560, B650, B725, B808, NDVI, CVI, *CI_RE_*, GNDVI, NDGI, NDRE, RVI	11	B808, CVI, NDGI, NDRE	-17
Texture	MEA, VAR, HOM, CON, DIS, ENT, SEC, COR	8	VAR, CON, SEC, COR	-17
Texture and VIs	Total VIs and Texture	19	NDRE, RVI, VAR, SEC, COR	-30

### 2.3 Prediction model

#### 2.3.1 Experimental flow

**Figure 3:**
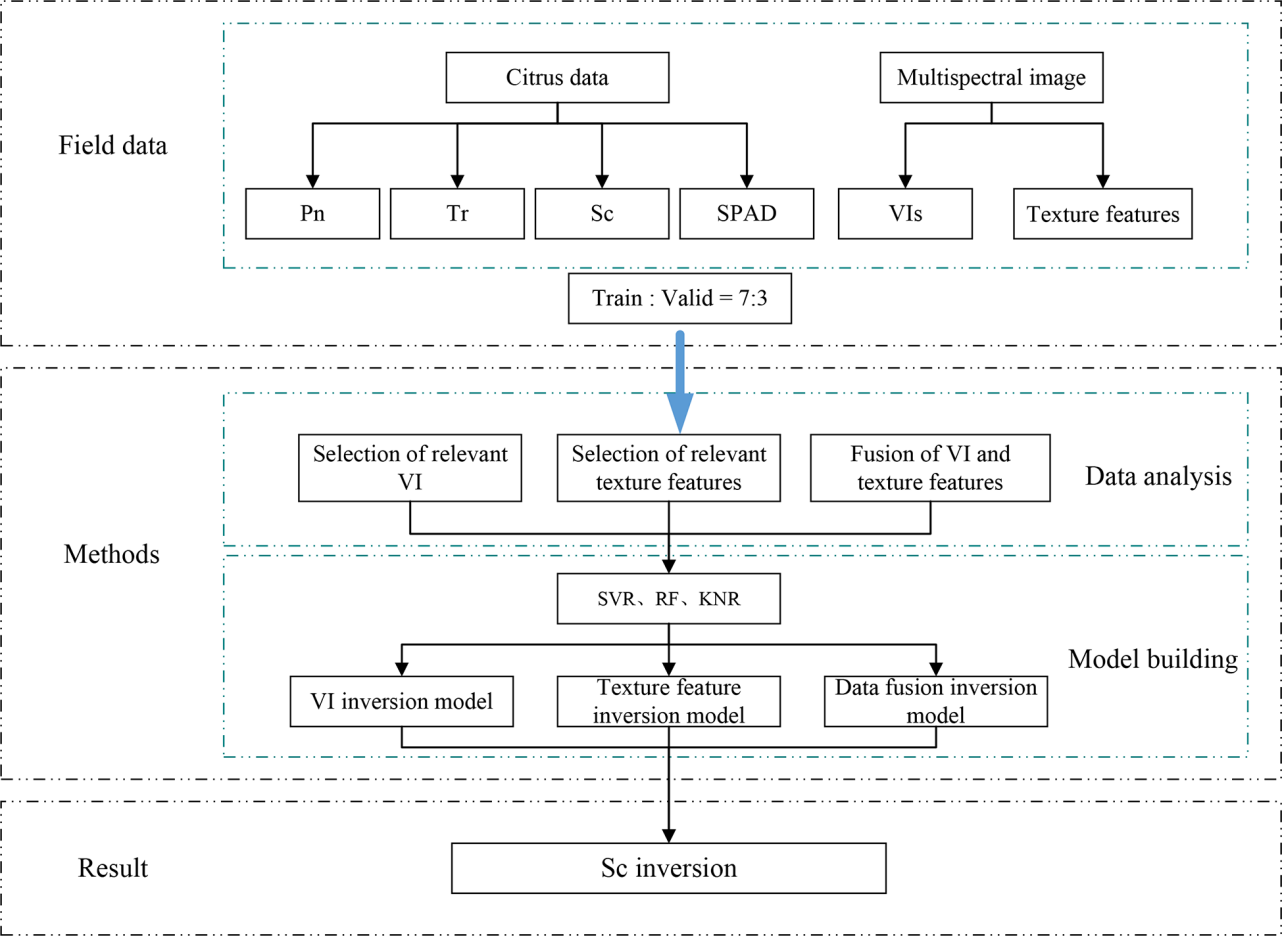
Experimental study flow chart.

A multispectral camera was used to collect data in different growth and development stages (fruit rapid expansion and fruit ripening) of the citrus trees, after which the images were processed. The experimental data were collected by relevant sensors and meteorological stations, mainly including citrus photosynthetic parameters, chlorophyll values, and meteorological data. In this study, mathematical statistics were used to conduct the statistical modeling, including machine learning methods (SVR, RF, and KNR). Finally, multispectral data were used to predict the photosynthetic parameters in the growing period of the citrus trees. The research flow chart is presented in **Figure 3**.

#### 2.3.2 Machine learning methods

Machine learning methods include SVR, RF and KNR, which are implemented through sklearn package in python3.7. When running regressions, the penalty factor C of SVR is 1, the radial basis function (RBF) is selected for the kernel function, and gamma is 2. The n_estimators for the number of trees in RF is 50. Other parameters are default. The KNR parameter is the default value.

#### 2.3.3 Model evaluation metrics

To evaluate the accuracy of the prediction model, the coefficient of determination (R^2^) and root mean square error (RMSE) were used to evaluate the model, where R^2^ reflects the fitting degree of the model to the measured values. The closer R^2^ is to 1, the better the fit. The RMSE is a commonly used evaluation index for regression models and reflects the dispersion degree of measured values near the regression line. The smaller the value of RMSE, the higher the model accuracy. The calculation formula is as follows:


(13) $$R^{2}=1-\frac{\sum_{1}^{m}{\left(yi-f\left(xi\right)\right)}^{2}}{\sum_{1}^{m}{\left(yi-\bar{yi}\right)}^{2}},$$

(14) $$RMSE=\sqrt{\frac{1}{m}\sum_{i=1}^{m}{\left(yi-f\left(xi\right)\right)}^{2}},$$

where *yi* represents the measured value of the sample, $\bar{yi}$ represents the mean value, and m represents the number of samples.

## 3 Results

### 3.1 Segmentation algorithm results

**Figure 4:**
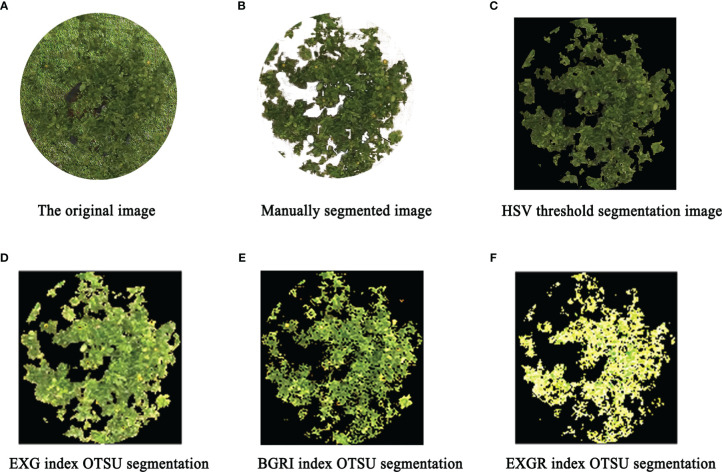
Partial segmentation effect diagrams (**A** is the original image; **B** is manually segmented image; **C** is HSV threshold segmentation image; **D** is EXG index OTSU segmentation; **E** is BGRI index OTSU segmentation; **F** is EXGR index OTSU segmentation).

The test results are listed in **Table 5**. For the complex citrus canopy leaf images in this study, the ACC and MCC levels of the HSV variable threshold segmentation method reached more than 80.0%, achieving accurate and effective segmentation. For the canopy images segmented by Otsu’s algorithm after visible light VI preprocessing, the segmentation effect varied with the VI. Although the ACC of VI IPCA was 96.27%, MCC was only 52.2%, which indicated that the segmentation effect of VI IPCA needed to be improved and that there was over-segmentation. Among the seven selected visible light VIs, EXG VI had the best segmentation effect, with ACC and MCC reaching nearly 80%. This is because the citrus canopy reflected the green band strongly, while the soil weakly reflected the green band. Moreover, EXG VI could distinguish the canopy and soil information more effectively, meaning that the segmentation effect of EXG VI was optimum in visible light VI (Senthilnath et al., 2017). It can be observed from **Table 5** that VIs with greater correlation with the green band exhibited relatively good segmentation effects, such as BGRI and EXGR. The partial segmentation results after VI treatment and HSV threshold calculation results are shown in **Figure 4**. The intersection over union (IOU) score is a standard performance measure for object class segmentation problems.

**Table 5:** ACC and MCC results of the segmentation algorithm.

Type	IOU	ACC	MCC
Principal Component Analysis Index (IPCA)	0.6757	0.9627	0.5220
HSV	0.8279	0.8426	0.8195
Excess Green (EXG)	0.7867	0.7912	0.7841
Blue-Green Ratio Index (BGRI)	0.5135	0.5851	0.4232
Green Minus Excess Red Index (EXGR)	0.4541	0.5062	0.3856
Blue-Red Ratio Index (BRRI)	0.3909	0.4450	0.2955
Normalized Green-Blue Difference Index (NGBDI)	0.2565	0.4359	-0.4229
Excess Red (EXR)	0.1361	0.1882	-0.2969

It can be seen from **Figure 4** that the four segmentation algorithms managed to divide the citrus canopy and soil background. According to the evaluation indicators of ACC and MCC, we adopted the HSV variable threshold segmentation method in this paper to segment the citrus canopy leaves, generate a vector file containing the pixel contour features of the citrus canopy, and then mask the multispectral images to obtain the canopy spectral data. About 100 multispectral images were used for analysis.

### 3.2 Effects of water stress on photosynthetic parameters of citrus trees

**Figure 5:**
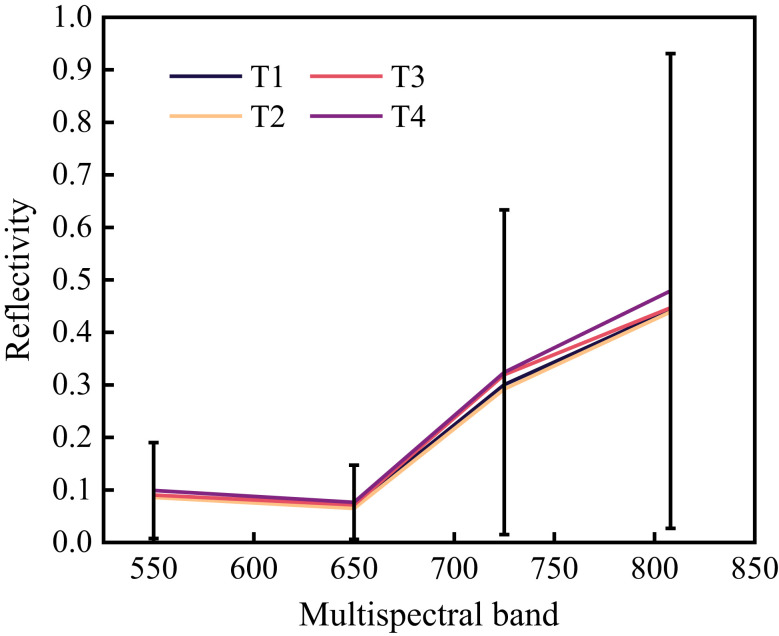
Reflectance curve of citrus canopy leaves.

Relevant studies (García-Tejero et al., 2012) suggested that T2 water treatment was the most suitable water gradient for citrus fruit growth, while T3 and T4 would produce water stress. The error bars in **Figure 5** represents the mean +/- standard deviation. It can be seen from **Figure 5** that water stress would affect the spectral reflectance of the citrus canopy, although it had almost no effect on the reflectance of the green (560 nm) and red (650 nm) bands. However, it had a significant effect on the reflectance of the red-edge (725 nm) and near-infrared (808 nm) bands. In the red-edge band (725 nm), the reflectance of T1 and T2 was low and the reflectance of T3 and T4 was high. In the near-infrared band (808 nm), the reflectance of T4 was significantly higher than that of T1, T2, and T3. Therefore, it is possible to identify whether citrus trees are in the water stress stage by judging the difference between the red-edge and near-infrared bands in the spectral reflectance of citrus canopy leaves.

**Figure 6:**
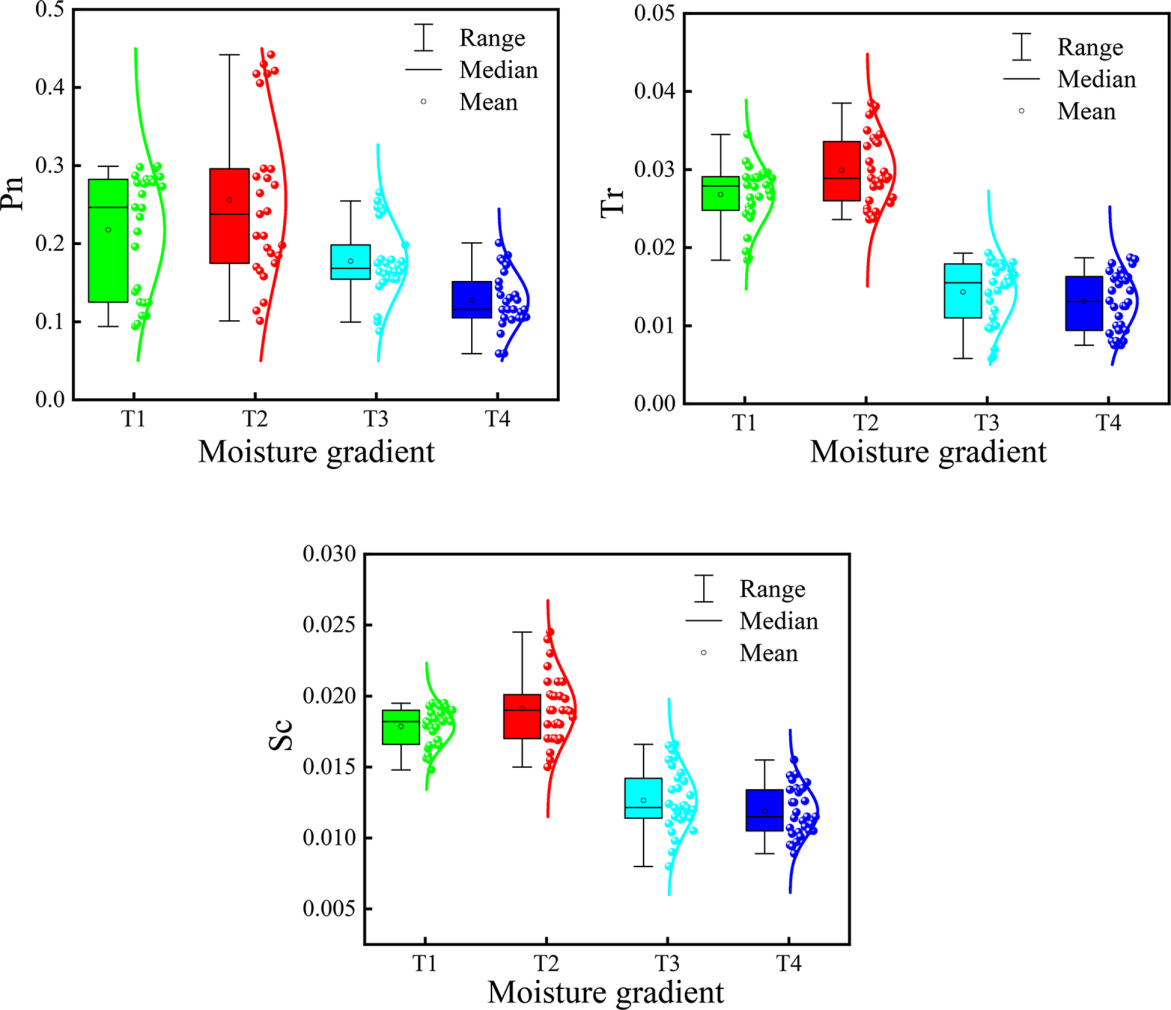
Distribution characteristics of Pn, Tr, and Sc under different water treatments.

**Figure 6** displays the distribution characteristics of Pn, Tr, and Sc under different water treatments. It can be seen from **Figure 6** that the Pn of leaves treated with T1 and T2 was higher than that of leaves treated with T3 and T4, indicating that water stress will have an impact on the Pn of citrus canopy leaves. This effect was positively correlated, with the higher the water stress degree, the lower the Pn. The Pn of the T1 treatment was not significantly different from that of the T2 treatment, indicating that the Pn of citrus canopy leaves would not change significantly under irrigation with sufficient water. The changes in transpiration rate and stomatal conductance of citrus canopy leaves under water stress were consistent. The Tr and Sc of leaves treated with T1 and T2 were higher than those treated with T3 and T4, which was consistent with the rule that water stress would affect the Tr and Sc of leaves. The higher the water stress, the lower the Tr and Sc.

### 3.3 Prediction model of citrus Sc based on vegetation index

**Figure 7:**
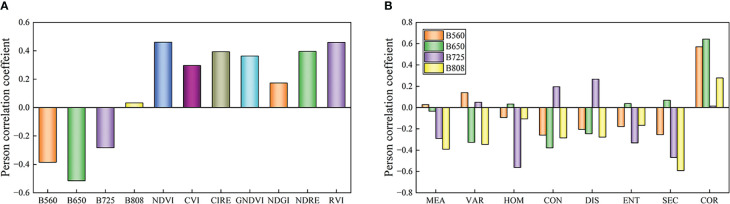
Pearson correlation coefficients.**(A)** Pearson correlation coefficient between vegatation index and Sc **(B)** Pearson correlation coefficient between image texture feature and Sc.

**Figure 8:**
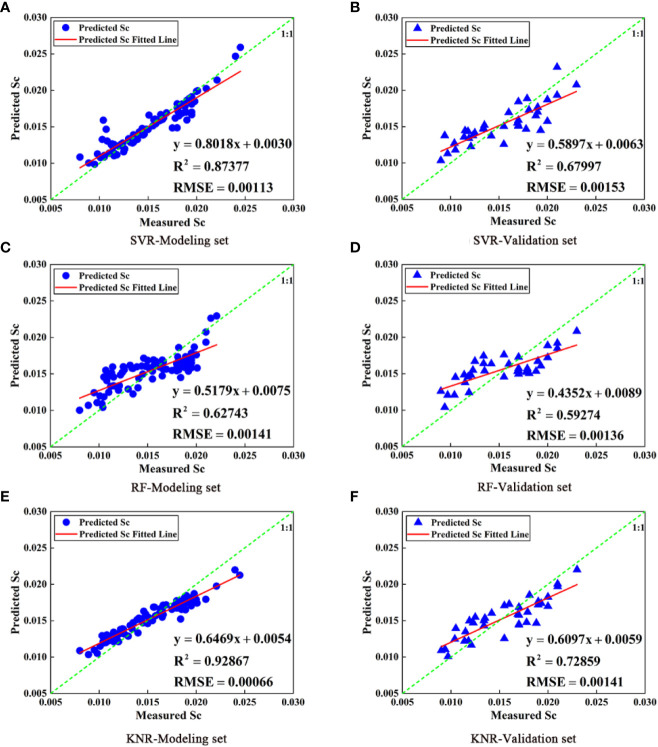
Comparison of Sc models for SVR, RF, and KNR prediction based on VI (**A**, **C**, **E** are modeling set; **B**, **D**, **F** are validation set).

**Figure 7A** displays the Pearson correlation coefficients between band reflectance and Sc, and between VIs and Sc. The VIs were positively correlated with Sc, while most citrus canopy reflectances were weakly and negatively correlated with Sc. Because most VIs and the band reflectance were correlated with Sc, full subset selection was used to screen all spectral band reflectance and VIs. The optimal index combination was selected as the independent variable (B808, CVI, NDGI, and NDRE), and Sc was taken as the dependent variable to construct the citrus Sc prediction model based on SVR, RF, and KNR. The modeling and validation sets of the Sc prediction model established by the SVR, RF, and KNR methods are depicted in **Figure 8**. Simultaneously, the prediction accuracy of the model (R^2^, RMSE) was verified by comparing the correlation between the predicted and measured Sc values of the model. The results indicate that the RF model achieved the lowest prediction accuracy (modeling set R^2^ = 0.62764, validation set R^2^ = 0.59274), and the KNR model has the highest prediction accuracy (modeling set R^2^ = 0.92867, validation set R^2^ = 0.72859). However, the RF model modeling set was closest to the validation set R^2^, meaning it had the highest stability. In the model based on SVR, RF, and KNR, the predicted Sc was high if the measured Sc was low, suggesting that when the measured Sc was low, the model accuracy would be correspondingly low.

### 3.4 Prediction model of citrus Sc based on texture features

**Figure 7B** displays the Pearson correlation coefficient between multispectral image texture features and Sc. Different colors and lengths in the histogram represent the different types of image texture features of the different bands (MEA, VAR, HOM, CON, DIS, ENT, SEM, and COR). The results indicate that the texture features of the four bands were correlated with Sc. Although the correlation between Sc and texture features of most bands was weak, the correlation between Sc and 808 nm texture features was high. **Figure 7B** shows that Sc was positively correlated with the 808 nm image texture feature (COR) and negatively correlated with other texture features in the four bands of the multispectral camera. Through the correlation analysis of image textures and Sc in different bands, it was evident that the image texture feature at 808 nm had the most significant correlation with Sc, while the image texture feature at other bands had a low correlation with Sc. The use of non-obvious image texture features would result in an increase in the RMSE of the model, which would reduce the prediction ability of the model.

**Figure 9:**
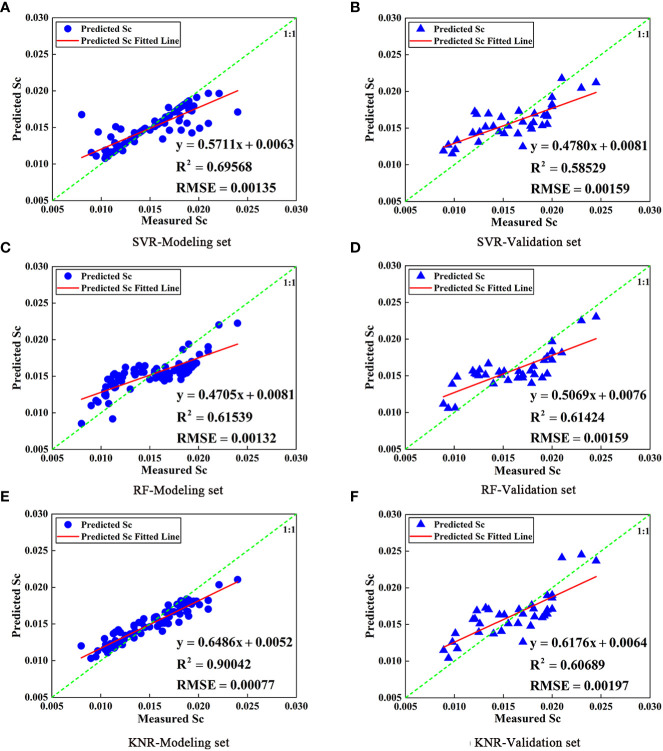
Comparison of Sc models for SVR, RF, and KNR prediction based on texture features (**A**, **C**, **E** are modeling set; **B**, **D**, **F** are validation set).

In this study, full subset selection was employed to screen the texture features of the 808 nm images. The optimal index combination was selected as the independent variable (VAR, CON, SEC, and COR), and Sc was taken as the dependent variable to construct the citrus Sc prediction model based on SVR, RF, and KNR. The predicted value was then compared with the measured value. The modeling and validation sets of the Sc prediction model established by the SVR, RF, and KNR methods are depicted in **Figure 9**. The results indicate that the Sc prediction model based on SVR, RF, and KNR methods and texture features was feasible. Succinctly, the accuracy of the three models was similar, and the Sc prediction model of the RF model achieved the highest accuracy (modeling set R^2^ = 0.61539, validation set R^2^ = 0.61424). However, the modeling set R^2^ (0.90042) of the KNR model was significantly larger than the validation set R^2^ (0.60689), indicating serious overfitting between the predicted and measured Sc. When the Sc of the citrus canopy was low, the Sc of the RF model was relatively high, which was similar to the results of the Sc prediction model based on VI and band reflectance. The accuracy of the Sc prediction model based only on texture features was lower than that based only on VI and band reflectance. This could either be because the accuracy of texture features is positively correlated with image resolution (affecting the extraction of texture features), or because the leaves of the citrus trees are relatively dense and the extraction of texture features is not obvious. Therefore, it is necessary to further study the combination of image texture features, VI, and band reflectance to construct the citrus Sc prediction model.

### 3.5 Prediction model of citrus Sc based on vegetation index and texture features

**Figure 10:**
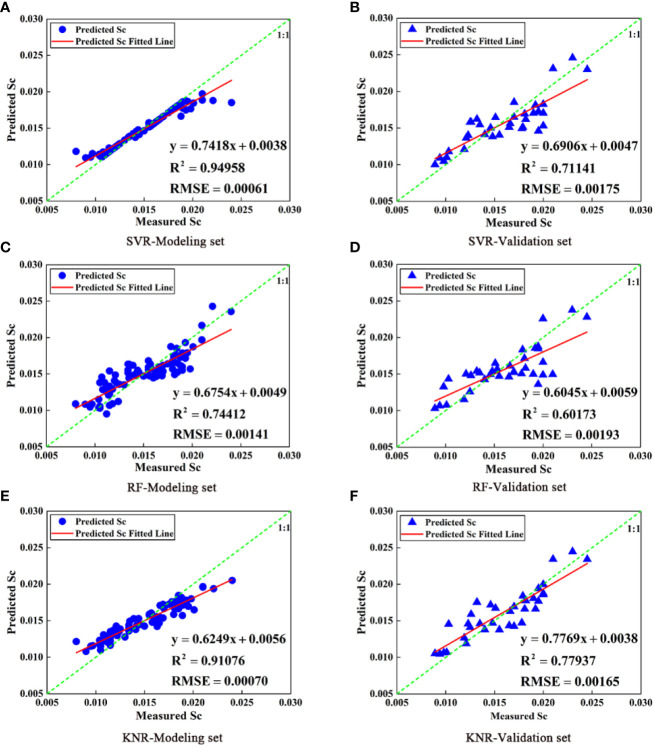
Comparison of Sc models for SVR, RF, and KNR prediction based on VI and texture features (**A**, **C**, **E** are modeling set; **B**, **D**, **F** are validation set).

In this study, the full subset selection method was used to screen the image texture features at 808 nm, the band reflectance, and VI to generate the best combination of image texture features, band reflectance, and VI. The optimum combination was selected as the independent variables (NDRE, RVI, VAR, SEC, and COR), and Sc was taken as the dependent variable to construct the citrus Sc prediction model based on SVR, RF, and KNR. **Figure 10** shows the relationship between the predicted and measured Sc values. The results suggest that KNR model achieved the highest accuracy in Sc prediction (modeling set R^2^ = 0.91076, RMSE = 0.00070, validation set R^2^ = 0.77937, RMSE = 0.00165). In addition, the Sc prediction model based on image texture features, VI, and band reflectance could solve the problem of large data prediction values when the Sc measurement values are small. It is evident from **Figure 10** that the R^2^ of the prediction models based on the SVR and KNR methods were both >0.7, and the accuracy was higher than that of the models based on VI or texture features. This suggests that the combination of VI and texture features can effectively improve the accuracy of prediction models. Therefore, the machine learning model based on image texture features, VI, and band reflectance can achieve higher accuracy than the machine learning model based on VI or texture features, and the Sc value of prediction is more accurate. The Sc of citrus decreases under water stress, and the Sc is different under different water treatments. However, the accuracy of multispectral prediction of Sc could reach 0.78, which can better predict Sc value. Therefore, whether crops are in a state of water stress could be judged by the value of Sc, so as to carry out corresponding irrigation treatment.

## 4 Discussion and conclusions

In this study, different water stress treatments were set for citrus trees, and Sc was measured during fruit growth and fruit maturity. Simultaneously, citrus Sc was predicted based on multispectral and machine learning methods. To reduce errors caused by the soil background, the threshold segmentation method was used to segment the citrus canopy leaves. In addition, differences between the SVR, RF, and KNR regression models were compared. The main conclusions are as follows:

1) In this study, the threshold segmentation method was used to process the citrus canopy, which could realize segmentation between the citrus canopy and the soil background. The ACC and MCC levels of the HSV variable threshold segmentation method were >80.0%. Moreover, the ACC and MCC of the canopy image segmented by Otsu’s algorithm after EXG preprocessing reached approximately 80%. Both methods achieved accurate and effective segmentation.
2) The citrus photosynthetic parameters (including Pn, Tr, and Sc) were all affected by different water supply treatments. The greater the degree of water stress, the lower the Pn, Tr, and Sc of the leaves (**Figure 6**). This result is consistent with the results of previous studies(Ballester et al., 2013).
3) Among the three machine learning models based on image texture features, VI, and band reflectance, the KNR model (NDRE, RVI, VAR, SEC, and COR) achieved the highest prediction accuracy. Compared with the KNR model based only on VI or image texture features, the validation set R^2^ of the KNR model based on combined variables was improved by 6.97% and 28.42%, respectively. Therefore, the multispectral visible light spectrum and texture features were combined to retrieve Sc, which provided a reference value for the judgment of citrus water stress.

Crop water stress is directly related to soil moisture. However, when the leaf coverage of the crop canopy is high, the information obtained by a multispectral camera is mainly about the crop canopy, which contains less soil information. Hence, the accuracy of using the canopy spectral information to estimate soil water content is low. This is because there is an indirect conversion process to diagnose crop water stress by retrieving soil moisture from crop canopy spectral information. Crop water status is associated with various indirect parameters that can be assessed by remote sensing, close observation, and spectral analysis techniques. Studies (Yang et al., 2021) have demonstrated that short-term water deficiency affects the growth process and results in stomatal closure, which ultimately leads to higher leaf temperature. Determining Sc is one of the methods used to quantify stomatal closure, and Sc is a physiological index that is widely used to detect crop water stress. Moreover, estimating Sc using canopy spectral information can directly diagnose crop water stress status. Currently, sensors are mainly used to measure Sc. Yang et al. (Yang et al., 2021) used photosynthetic apparatus to measure the Sc of the Chinese cabbage heart to judge its water stress degree. However, the measurements were time-consuming and laborious and were not widely applicable. Kandylakis et al. (Kandylakis et al., 2020) used aerial short-wave infrared and multispectral data to measure grape water stress, and they achieved high levels of accuracy. Their conclusions were similar to the results of this study, which showed that the Sc prediction accuracy could reach more than 0. 8 by combining texture characteristics and vegetation index.

There are more than 100 types of multispectral VIs, and how to select a suitable one for research purposes remains a significant problem. Currently, most studies select some VIs and take them all as model inputs. Although this can improve the accuracy of some prediction models, it can significantly improve the dimension of the model data, and the computation time is increased. In this study, the full subset selection method was used to combine variables and select the optimal combination of variables. This ensured that the R^2^ value of the model was the highest or the BIC value was the lowest, achieving the purpose of reducing the input variables of the model. Zhang et al. (Zhang et al., 2021a) used the full subset selection method to screen the multispectral VI and determined the optimal variable combination of VIs to realize remote sensing estimation of soil salinity. Their conclusion was similar to the result of this study, which achieves the purpose of variable combination optimization.

Many studies have only used VI to retrieve canopy information of crops. While this can achieve the effect of basic prediction, the method has limitations. When crops are subjected to water stress, the canopy structure will change, such as mild leaf wilting. Furthermore, this model only uses VI as the input, which can only be reflected when the leaf color changes significantly. Some studies(Dube and Mutanga, 2015; Schumacher et al., 2016) have shown that image texture features can retrieve crop biomass and achieve a better prediction effect than VIs, although most studies have focused on forests. Compared with previous studies (Liu et al., 2019), this study did not find that single texture features could retrieve Sc more accurately than VIs, which may be because citrus leaves are relatively dense and the extracted texture features are regional features of multiple leaves. Furthermore, the image texture features of leaves are related to image resolution, where the higher the resolution, the clearer the extracted image texture features. This study has revealed that the addition of texture features can improve the accuracy of prediction and provide more accurate Sc estimation compared with models based only on VIs. Therefore, the Sc prediction model using the combination of VIs and texture features as input has a better prediction effect than the single variable input model.

The VI can easily be influenced by the soil background when obtaining crop canopy information. When the crop coverage is low, the VI is prone to light saturation. In this study, a threshold segmentation method was used to segment multispectral images and extract citrus canopy leaf data, and the segmentation effect could reach approximately 80%. Long et al. (Long et al., 2020) used Otsu’s method to segment multispectral images of a maize plant canopy and achieved the effect of preliminary segmentation of flowerpots and standard plates, which was similar to the results of this study. The results suggest that the segmentation method used in this study can reduce the influence of soil background and reduce experimental error. However, we only used the color threshold segmentation method and did not employ neural networks for segmentation. On the one hand, because the color difference between the citrus canopy and soil background is large, the segmentation effect can be achieved by threshold segmentation. On the other hand, the leaves of the citrus canopy are small and the edges of the leaves are complex, which is not conducive to segmentation by neural networks. Moreover, there are problems such as complex data annotation.

While the machine learning method used in this study was effective in estimating citrus Sc, there were some limitations. First, there is a correlation between texture features and image resolution, whereby the higher the resolution, the clearer the texture features. However, in this study, we did not consider the effect of different resolutions on texture features, which requires further study. Second, when collecting spectral data, we did not consider whether factors such as the solar height and the citrus leaf angles would affect the scale of reflectance. Finally, we only studied the water stress in the growth stage of citrus fruits, not in the whole growth stage. To determine whether the model is applicable to other stages would require further study.

Considering the data characteristics of citrus multi-spectral image, such as small amount of data and strong nonlinear, it was unable to meet the requirements of convolutional neural network for large-scale data sets. Therefore, the traditional machine learning method with advantages of classification and inversion in small sample data was selected and the algorithms were compared at the same time. In the future, more advanced machine learning methods will be considered to improve the accuracy of inversion.

In this study, we through the two aspects to solve the fitting problem. First, the full subset screening method assumes that there are N independent variables in the regression analysis, and any M(1≤M≤N) independent variables in N establish regression equations with dependent variable Y respectively. A model fitting 1 ~ N prediction variables was obtained. Among the 1 ~ N models, N optimal models are selected according to the maximum criterion of the adjusted coefficient of determination. The adjusted coefficient of determination considers the influence of the number of independent variables and the number of samples. With the increase of the model’s independent variables, its value will not increase, which reduces the overfitting phenomenon. Second, the method of cross validation was added to the algorithm in this paper, and the data set was divided into 7:3, including two training sets and one test set. All data will be involved in training and prediction, effectively avoiding overfitting and fully reflecting the idea of crossover. In addition to the above two methods, we could suppress overfitting problems by increasing data or thinking about Regularization in the future.

This study proved that multispectral VI and texture features can be used to judge the water stress in citrus trees. However, the relationship between texture features and image resolution can be further studied to determine the influence of different resolutions on the research results. Although spectral data can effectively predict Sc, it is necessary to further consider the influence of solar height angle, citrus leaf angle, and other factors during data collection to enhance the reliability of this study. Finally, it is recommended that future research studies the whole growth cycle of citrus trees to realize the determination of water stress in the whole cycle.

## Data availability statement

The raw data supporting the conclusions of this article will be made available by the authors, without undue reservation.

## Author contributions

JX, and YC: conception and design. ZY, and JW: formal analysis. GL, PG, and ZS: visualization. JX, YC, and DS: wring and revision of the manuscript. DY, WW, and JL: funding acquisition and study supervision. All authors contributed to the article and approved the submitted version.
